# School Hygiene

**Published:** 1905-02-18

**Authors:** 


### The Hospital.

A Journal of

The Medical Sciences and Hospital Administration.

Vol. XXXVII.—No. 960. FEBRUARY 18, 1905.

### School Hygiene.

The Conference on School Hygiene, which we lately announced as about to be held in the buildings of the University of London, and under the direction of the Royal Sanitary Institute of Great Britain, afforded opportunities for a considerable amount of discussion of the principal points involved. It will, we hope, at least be useful in clearing the ground for the larger Congress, to be held in 1907, of which it was declared to be the precursor; but it can hardly be said to have yielded any large increase of the pre-existing knowledge of the subjects to be considered. The addresses, delivered by Sir Arthur Rücker, Sir William Anson, Sir Aston Webb, and Sir William J. Collins, were all as instructive as might have been expected from the eminent abilities of the gentlemen concerned in their production; but they do not appear to have emphasised with sufficient force the desirability of radical changes either in school management or in the health of scholars. The one disclosure which might be described by a certain section of the Press as "sensational" was that made by a Japanese lady, Miss Miyakama, who described to the Conference the arrangements which she had seen at a truant school in Liverpool, where 20 successive male truants were permitted or compelled to "wash" in succession in a single supply of hot water, and where the hot water in which certain girls had been washed was "very black." If we may assume the propriety of the course pursued with the boys, we shall be compelled to approve also that which was pursued with the girls. The Liverpool authorities are evidently anxious to conform to the precept of the angel in the Apocalypse, "He which is filthy, let him be filthy still," as well as with the more recent opinion of Mrs. Poyser, that women were made (not filthy, but "fullish") in order that they might "match the men."

The question almost naturally suggests itself whether, in lieu of conferences, it might not be possible to obtain in schools some modicum of sensible and concerted action—some small beginning, at least, of a strife against admitted evils. Would it not be possible to establish some system of visitation by school managers of such a kind that when the visitor, on entering a class-room, was irresistibly compelled by the state of the contained atmosphere to say "Phew," he or she should have authority to clear the room for five or ten minutes, to disperse the children through the playground, to open all the doors and windows, and generally to bring about an air-supply proper for the respiration of living creatures? Would it not be possible to give such orders that the same course would be pursued at regular intervals even when no visitor came to enforce it? In proportion as the exclamation presupposed was called forth not only by the presence of carbon dioxide, but also by the stench arising from dirty bodies and from foul clothing, would it not be possible for the managers, as a body, to aim at some improvement in the habits of the people by whom the scholars were supplied, not proceeding, of course, after the manner of Mrs. Pardiggle, but by trifling rewards and by judicious inducement? Would it not be possible for teachers to learn to recognise the signs of under-feeding or of mental or bodily fatigue, and to recognise, at the same time, that the exercise of pressure upon children by whom these signs were displayed would only defeat its own object and render their last state worse than their first? We are strongly of opinion that a rectification of many of the hygienic evils incidental to schools as now conducted might be brought about by the exercise of a modicum of common sense on the part of teachers and of local authorities, and that the invocation of Hercules, in the shape of a Government inspector, or of a County Medical Officer of Health, might thus be rendered unnecessary. The chief impediment is, of course, the tradition hitherto fostered by the Education Department that the efficiency of a school is to be measured by the steady ascent of the pupils through successive standards to be tested by examination; the substitution, that is to say, of artificial tests for those natural ones which would be afforded by cleanliness, by behaviour, and by intelligence. The teacher too often fancies himself compelled to work for conditions other than the best, in order to satisfy the Procrustean requirements upon which the school may be dependent for a "grant" necessary to its existence. If the best possible work is to be expected from him, and if he is to exercise intelligent control over the hygienic conditions of the children under his charge, he ought not only to possess full discretion in many matters of routine, but he ought also to be considered accountable for its complete and judicious exercise. His duties afford him unlimited opportunities of observing the differences which distinguish one child from another, and it should be his business to meet these differences by corresponding differences of treatment and of requirement. The first, and eminently successful, headmaster of the great elementary school at Edinburgh, which, with others more advanced in succession to it, was founded thirty or forty years ago with the funds afforded by the disestablishment of the old "Hospitals" of the city, was accustomed to say that he had one great principle—namely, that there were no bad boys and no boobies. The exceptions were apparent only, and their cases were always to be met by exceptional treatment.

